# Incidence and risk factors of cardiovascular disease among population aged 40–70 years: a population-based cohort study in the South of Iran

**DOI:** 10.1186/s41182-023-00527-7

**Published:** 2023-06-12

**Authors:** Abbas Rezaianzadeh, Leila Moftakhar, Mozhgan Seif, Masoumeh Ghoddusi Johari, Seyed Vahid Hosseini, Seyed Sina Dehghani

**Affiliations:** 1 Colorectal Research Center, Shiraz University of Medical Science, Shiraz, Iran; 2 Student Research Committee, Shiraz University of Medical Sciences, Shiraz, Iran; 3 Department of Epidemiology, Faculty of Biostatistics, School of Health, Shiraz University of Medical Sciences, Shiraz, Iran; 4 Breast Diseases Research Center, Shiraz University of Medical Sciences, Shiraz, Iran; 5 School of Medicine, Shiraz University of Medical Sciences, Shiraz, Iran

**Keywords:** Cardiovascular diseases, Incidence, Risk factors, PERSIAN cohort–Cox regression

## Abstract

**Background:**

Cardiovascular diseases are the main cause of mortality in the world. This study aimed to estimate the incidence and identify the risk factors of these diseases.

**Methods:**

This prospective cohort study was performed on 9442 individuals aged 40–70 years in Kharameh, a city in the South of Iran, in 2015–2022. The subjects were followed up for 4 years. The demographic information, behavioral habits, biological parameters, and history of some diseases were examined. The density incidence of cardiovascular disease was calculated. The log-rank test was calculated to assess the cardiovascular incidence difference between men and women. Simple and multiple Cox regression with Firth's bias reduction method were used to identify the predictors of cardiovascular disease.

**Results:**

The mean ± SD age of the participants was 51.4 ± 8.04 years, and the density incidence was estimated at 1.9 cases per 100,000 person-day. The log-rank test showed that men had a higher risk of cardiovascular disease than women. The Fisher's exact test showed a statistically significant difference between the incidence of cardiovascular diseases in different age groups, education levels, diabetes, and hypertension in men and women. The results of multiple Cox regression revealed that with increasing age, the risk of developing CVDs increased. In addition, the risk of cardiovascular disease is higher in people with kidney disease (HR_adj_ = 3.4, 95% CI 1.3 to 8.7), men (HR_adj_ = 2.3, 95% CI 1.7 to 3.2), individuals with hypertension (HR_adj_ = 1.6, 95% CI 1.3 to 2.1), diabetics (HR_adj_ = 2.3, 95%c CI 1.8 to 2.9), and alcohol consumption (HR_adj_ = 1.5, 95% CI 1.09 to 2.2).

**Conclusions:**

In the present study, diabetes, hypertension, age, male gender, and alcohol consumption were identified as the risk factors for cardiovascular diseases; three variables of diabetes, hypertension and alcohol consumption were among the modifiable risk factors, so if they were removed, the incidence of cardiovascular disease could greatly reduce. Therefore, it is necessary to develop strategies for appropriate interventions to remove these risk factors.

## Background

Cardiovascular diseases (CVDs) are among the most common non-communicable diseases [1]. They are the leading cause of death worldwide, and it is estimated that out of 55 million deaths in 2017, 17.7 million were related to CVDs [2, 3]. In addition, it is predicted that this figure will reach 23.6 million in 2030 [4, 5]. According to the Global Burden of Diseases (GBD) report in 2019, total cases of CVDs doubled from 1990 to 2019 and increased from 271 to 532 million. In addition, Disability Adjusted Life Years (DALYs) increased from 17.7 million to 34.4 million [6]. The report on the burden of diseases in 2015 introduced Iran as one of the countries with the highest rates of CVDs in the world due to having more than 9000 cases of CVDs per 100,000 population. In addition, it has been stated that the mortality rate due to these diseases has increased in Iran. Moreover, CVDs have important clinical consequences, cause premature death, and reduce the quality of life [5, 7].

There are several risk factors for CVDs that are generally divided into two categories: non-modifiable (age, sex, race, and family history) and modifiable (diabetes, lipid profile, hypertension, alcohol consumption, smoking, inadequate physical activity, inappropriate diet, and obesity) [8, 9]. Behavioral, environmental, and social factors are the other risk factors for CVDs [6]. Global evidence shows that by controlling and managing the modifiable risk factors, up to 90% of cases of CVDs can be reduced [5]. Therefore, appropriate identification of individuals with these risk factors and those susceptible to CVDs is an important step in controlling these diseases [10].

Epidemiological studies have played an important role in understanding the risk factors of CVDs [6]. Nevertheless, health service planning units in Iran do not yet have accurate estimates of the current status of CVDs regionally [11]. At the same time, we need to accurately estimate the incidence rate and risk factors of CVDs in each region to determine appropriate strategies for preventing and controlling these diseases [5]. In addition, most studies in this field have been performed on a specific group of patients, and information on the general population has not been extensively reviewed. Therefore, this study was conducted to investigate the incidence rate and identify the risk factors affecting the incidence of CVDs in the population aged 40–70 years in Kharameh in the South of Iran to help health policymakers to develop preventive guidelines.

## Methods

### Kharameh cohort study design

The present prospective cohort study was conducted based on the data of the Kharameh cohort study, which is part of a large Prospective Epidemiological Studies in Iran (PERSIAN) launched in 2014. Other details are explained in the Persian cohort study [12]. The target population of the Kharameh cohort study includes all individuals aged 40–70 years. At first, all these individuals were invited to participate in the study. Finally, 10,663 subjects (97.3% participation rate) participated in the study after they signed the informed consent forms.

**Fig. 1:**
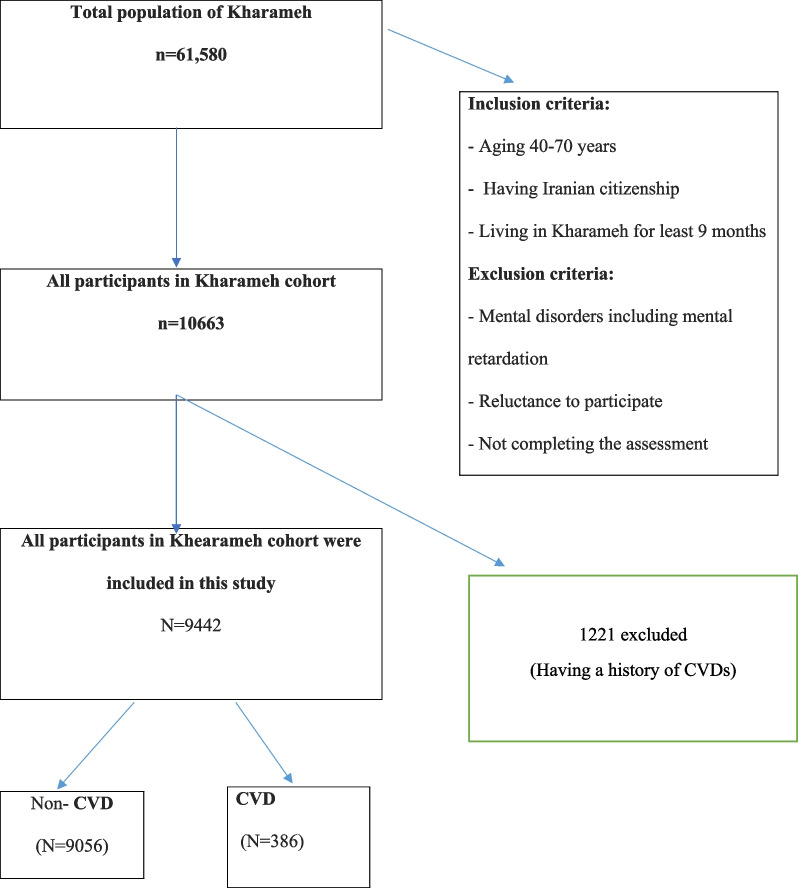
Flow chart of the study population

The inclusion criteria for the Kharameh cohort study were age 40–70 years and at least 9 months of residence in Kharameh to allow time to adapt to the environment and culture [13]. Exclusion criterion was the individuals with mental retardation or mental disorders who could not participate in the study. In addition, the exclusion criteria of this study were having a history of CVDs, heart attacks, and stroke. At first, there were 1221 people; after some of them were excluded, 9442 subjects were followed up in the present study (Fig. 1).

The baseline data of the Kharameh cohort study were collected from March 2015 to March 2017, and the information about the incidence of CVDs in individuals was collected during four stages of the follow-up in 2018, 2019, 2020, and 2021. Trained experts collected the participants' demographic information and behavioral habits through face-to-face interviews; physicians of the Kharameh cohort team recorded their clinical information. Questionnaires related to the PERSIAN cohort study, which had previously been validated, were used to collect the data. The history of chronic diseases in individuals was recorded by their self-declaration and review of medical records by physicians.

### Demographic and clinical data of the study patients

In this study, data related to the demographic characteristics of individuals and some of their behavioral habits were used. This information included age, sex, marital status, education, place of residence, having a job, Body Mass Index (BMI), waist circumference, hip circumference, alcohol consumption, smoking, and socioeconomic status (SES). In this study, having a job was defined as working at least 8 h per week at the time of enrollment visit. In addition, alcohol consumption and smoking were considered as drinking approximately 200 ml of beer or 45 ml of liquor, once per week for at least 6 months, and smoking at least 100 cigarettes during lifetime, respectively.

The history of diseases such as diabetes, hypertension, fatty liver disease, and chronic kidney disease was also examined. In addition, fasting blood sugar (FBS), low-density lipoprotein (LDL), triglyceride (TG), and high-density lipoprotein (HDL) were assessed. To assess the SES of the individuals, we completed the PERSIAN cohort questionnaires related to the socioeconomic information of individuals, and the collected variables related to SES were analyzed using Principal Component Analysis (PCA) to identify the components for grouping related individuals into different SES categories. Accordingly, the participants were divided into four classes: low, moderate, high, and very high [14].

Weight was measured without shoes and with light clothing using a SECA scale (made in Germany), and height was measured using a standard measuring tape. BMI was calculated by dividing the body weight (kilogram) by height squared (meter). Accordingly, the participants were divided into four groups: underweight (less than 18.5 kg/m^2^), normal (18.5 to 24.9 kg/m^2^), overweight (25 to 29.9 kg/m^2^), and obese (over 30 kg/m^2^) [15]. For the laboratory experiments, the individuals were requested to fast for 12 defined as a history of diabetes, treatment of diabetes, or fasting blood sugar above 126 mg/dL. Blood pressure was measured from the participants' left arms using a standard calibrated sphygmomanometer (Reister Model, Germany) after a 5-min rest in a sitting position. It was measured twice with an interval of 10 min, and the mean was recorded.

### Cardiovascular disease

In this study, individuals were followed for 4 years from 2018 to 2021, and in each follow-up period, we initially recorded the incidence of CVDs according to their self-declaration. Then, their medical records were reviewed by physicians, and if confirmed, they were registered as a new case of CVDs. CVDs in this study included coronary heart disease, cerebrovascular disease, rheumatoid arthritis, myocardial infarction, stroke, and heart valve disease.

### Statistical analysis

In the present study, the dependent variable was the time to event of CVDs, from the time of enrollment until the event of CVDs. In addition, individuals were considered right censors if they did not have the event of CVDs until the end of the study. Quantitative and qualitative variables were described with the mean (standard deviation) and number (percentage). The Kolmogorov–Smirnov test determined the normality of quantitative variables. The difference between the mean of quantitative variables and the levels of qualitative variables between the two groups with and without CVDs was assessed using an independent *t* test, Mann–Whitney, and Fisher's exact test. The density incidence rate was calculated in terms of person-day unit, which is the actual number of days that individuals are at risk of CVDs. We summed the days of observation which started from the participant's enrollment to the date of the event of CVDs or the end of the study. For survival analysis, the Kaplan–Meier curve for CVDs was plotted, and the log-rank test was calculated to compare the risk of CVDs curve between men and women. Finally, simple Cox regression with Firth's bias reduction method was used to identify the risk factors of CVDs. To control the confounders, we entered all variables with a *p* value less than 0.2 into multiple Cox regression. The association was also reported with Hazard Ratio (HR) with a 95% confidence interval (CI). Firth's method was used in the Cox proportional hazards framework to develop and validate a prediction model for rare event survival data (heavily censored). All analyses were carried out using software R version 4.1.2, the "Coxphf" package, and STATA software version 12.

## Results

In the present study, 9442 individuals aged 40–70 with a mean age of 51.47 ± 8.04 were followed up for 19,744,954 person-days. During this period, 386 new cases of CVDs were observed. The density incidence was 1.9 cases per 100,000 persons-days. Women and the illiterate accounted for 55.6% and 50.7% of the participants, respectively. Most participants were married (89.7%), and 59% were overweight and obese (Table 1).

**Table 1:** Distribution of demographic characteristics and behavioral habits in a population aged 40–70 years of Kharameh cohort study

Variable	Category	Total *N* (%)	Men *N* = 4192 (44.4%)			Women *N* = 5250 (55.6%)		
			Without CVD *N* = 3956 (94.4)	With CVD *N* = 236 (5.6)	*p* value	Without CVD *N* = 5100 (97.1)	With CVD *N* = 150 (2.9)	*p* value
Age	40–50	4556 (48.3)	1876 (97.5)	48 (2.5)	< 0.01	2607 (99)	25 (1)	< 0.01
	50–60	3238 (34.3)	1449 (92.8)	112 (7.2)		1625 (96.9)	52 (3.1)	
	60–70	1648 (17.4)	631 (89.2)	76 (10.8)		868 (92.2)	73 (7.8)	
Having Job	No	4400 (46.6)	502 (91.6)	46 (8.4)	< 0.01	3741 (97.1)	111 (2.9)	0.92
	Yes	5042 (53.4)	3454 (94.8)	190 (5.2)		1359 (97.2)	39 (2.)	
Married status	Single	166 (1.8)	21 (100)	0	0.12	144 (99.3)	1 (0.7)	< 0.01
	Married	8472 (89.7)	3907 (94.3)	232 (5.7)		4226 (97.5)	107 (2.5)	
	Divorced	804 (8.5)	28 (87.2)	47 (12.5)		730 (94.5)	42 (5.5)	
Socioeconomic status	Low	2372 (25.1)	820 (94.6)	4 (5.4)	0.03	1455 (96.7)	50 (3.3)	0.13
	Moderate	2636 (27.9)	855 (92.9)	65 (7.1)		1660 (96.7)	56 (3.3)	
	high	2229 (23.7)	812 (93.7)	55 (6.3)		1332 (97.8)	30 (2.2)	
	Very high	2205 (23.3)	1469 (95.5)	69 (4.5)		653 (97.9)	14 (2.1)	
Education	Illiterate	4793 (50.7)	1461 (92.3)	122 (7.7)	< 0.01	3082 (96)	128 (4)	< 0.01
	Primary school	2430 (25.7)	1064 (95.2)	54 (4.8)		1296 (98.8)	16 (1.2)	
	Secondary school	1052 (11.1)	640 (95.5)	30 (4.5)		378 (99)	4 (1)	
	Diploma and University	1167 (12.5)	791 (92.3)	30 (3.7)		344 (99.5)	2 (0.5)	
Cigarette smoking	No	7083 (75.1)	1881 (94.3)	113 (5.7)	0.94	4954 (97.3)	135 (2.7)	< 0.01
	Yes	2359 (24.9)	2075 (94.4)	123 (5.6)		146 (90.7)	15 (9.3)	
Alcohol consumption	No	8924 (94.5)	3478 (94.5)	201 (5.5)	0.21	5096 (97.2)	149 (2.8)	0.13
	Yes	518 (5.5)	478 (93.2)	35 (6.8)		4 (80)	1 (20)	
Location	Urban	3379 (35.7)	1503 (94.4)	89 (5.6)	0.94	1753 (98.1)	34 (1.9)	< 0.01
	Rural	6063 (64.3)	2453 (94.4)	147 (5.6)		3347 (96.6)	116 (3.4)	
BMI	Underweight	380 (4)	279 (96.8)	9 (3.2)	0.21	88 (95.6)	4 (4.4)	0.12
	Normal	3496 (37)	194 (94.3)	117 (5.7)		1381 (96.4)	52 (3.6)	
	Overweight	3913 (41.5)	1439 (94.2)	89 (5.8)		2325 (97.5)	60 (2.5)	
	Obese	1653 (17.5)	292 (93.3)	21 (6.7)		1306 (97.4)	34 (2.6)	
Chronic kidney disease	No	9411 (99.7)	3940 (94.4)	233 (5.6)	0.08	5089 (97.1)	149 (2.9)	0.29
	Yes	31 (0.3)	16 (84.2)	3 (15.8)		11 (91.6)	1 (8.4)	
Hypertension	No	7634 (80.8)	3568 (95.2)	181 (4.8)	< 0.01	3808 (98)	77 (2)	< 0.01
	Yes	1808 (19.2)	388 (87.6)	55 (12.4)		1292 (94.7)	73 (5.3)	
Diabetes mellitus	No	8182 (86.6)	3640 (95)	188 (5)	< 0.01	4271 (98.1)	83 (1.9)	< 0.01
	Yes	1260 (13.4)	316 (86.9)	48 (13.1)		829 (92.5)	67 (7.5)	
Fatty liver	No	8383 (88.8)	3702 (94.3)	14 (5.7)	0.89	4331 (97.1)	128 (2.9)	1
	Yes	1059 (11.2)	254 (94.8)	222 (5.2)		769 (97.2)	22 (2.8)	

Based on the results presented in Table 1, the incidence of CVDs was higher in the older age groups for both sexes (*p* < 0.01). The incidence of CVDs was associated with marital status of women (*p* < 0.01) and with the SES level of men (*p* = 0.04). In addition, the incidence of CVDs was associated with education level, status of diabetes, and high blood pressure in both men and women. (*p* < 0.01).

There was a statistically significant difference between the FBS, LDL, TG, and waist and hip circumference in the two groups of women with and without CVDs. In addition, there was a significant difference between the mean of LDL in men with and without CVDs (Table 2).

**Table 2:** Distribution of anthropometric characteristics and laboratory experiments in a population of 40–70 years

Variable	Men			Women		
	Without CVD Mean ± S.D	With CVD Mean ± S.D	*p* value	Without CVD Mean ± S.D	With CVD Mean ± S.D	*p* value
TG	129.18 ± 75.33	132.42 ± 78.52	0.52	128.87 ± 73.87	148.03 ± 100.63	< 0.01
LDL	104.57 ± 27.29	110 ± 28.54	< 0.01	105.61 ± 27.58	125.35 ± 166.24	< 0.01
HDL	46.67 ± 12.02	47.63 ± 12.74	0.23	48.53 ± 12.95	49.71 ± 13.09	0.27
FBS	97.17 ± 30.29	100.79 ± 33.45	0.07	99.44 ± 33.49	118.47 ± 59.34	< 0.01
Hip circumference	99.04 ± 7.25	99.53 ± 7.27	0.31	102.31 ± 8.99	100.09 ± 9.5	< 0.01
Waist circumference	97.21 ± 11.79	97.98 ± 12.06	0.43	92.43 ± 11.87	94.73 ± 10.7	< 0.01

*CVD* cardiovascular disease, *TG* Triglyceride, *LDL* low-density lipoprotein, *HDL* high-density lipoprotein, *FBS* fasting blood sugar

**Fig. 2:**
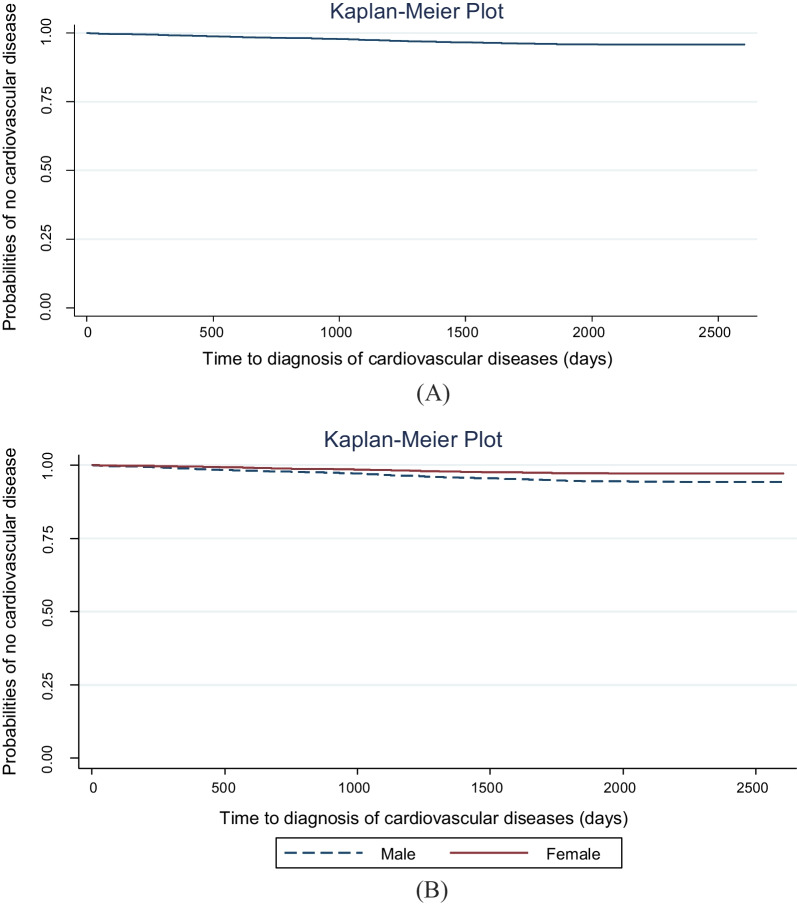
Kaplan–Meier curve of the time to first diagnosis of cardiovascular diseases in a population of 40–70 years in the Kharameh cohort study (**A**), and by gender (**B**)

Figure 2A shows the Kaplan–Meier survival curve for all individuals, and Fig. 2B displays the Kaplan–Meier survival curve by gender. It was observed that the risk of developing CVDs was higher in men than in women. The log-rank test (*p* < 0.01) also revealed that the difference between women and men was statistically significant.

In addition, we performed simple Cox regression to identify the predictors of CVDs. The results of simple Cox regression showed a statistically significant relationship between the variables of sex, age, marital status, having job, alcohol consumption, smoking, history of diabetes, chronic kidney disease, hypertension, education, location, hip circumference, waist circumference, TG, LDL, and FBS with the risk of developing CVDs. Then, we examined the correlation between smoking and alcohol variables, LDL and TG, LDL and HDL, as well as waist circumference and hip circumference. A high correlation was observed only between waist circumference and hip circumference (*ρ* = 0.8. *p* < 0.0001). For this reason, we did not enter the waist circumference variable into the First Cox multiple regression model. Finally, after performing multiple Cox regression to control the confounders, we found that with increasing age, the risk of developing CVDs increased, so that the risk of developing CVDs in individuals was 2.4 times higher in the age group of 50–60 years and 3.7 times higher in individuals aged 60–70 years than the 40–50-year-old subjects. The risk of developing CVDs in men was 2.3 times higher than in women (HR_adj_: 2.3, 95% CI 1.7–3.2); in individuals with chronic kidney disease, it was almost 3.4 times higher than in participants without chronic kidney disease. (HR_adj_: 3.4, 95% CI 1.3–8.7). People with diabetes were 2.3 times more likely to develop CVDs than non-diabetics. The risk of CVDs in the subjects with hypertension was 68% higher than those without hypertension; also, in subjects who consumed alcohol, it was 58% higher. There was also a small but statistically significant positive relationship between LDL and the risk of CVDs (Table 3).

**Table 3:** Predictive factors of incidence of CVD in Kharameh population according to multivariable Cox regression with Firth's bias reduction method analysis

Variable	Class	Simple Cox regression		Multiple Cox regression	
		HR (95%CI)	*p* value*	HR_adj_ (95%CI)	*p* value*
Age	40–50	1		1	
	50–60	3.2 (2.4,4.2)	< 0.01	2.4 (1.8,3.3)	< 0.01
	60–70	5.7 (4.3,7.6)		3.7 (2.7,5.2)	
Sex	Female	1		1	
	Male	2.02 (1.6,2.4)	< 0.01	2.3 (1.7,3.2)	< 0.01
Having Job	No	1		1	
	Yes	1.2 (1.05,1.5)	0.013	1.1 (0.9,1.5)	0.2
Married status	Single	1	0.001	1	0.32
	Married	4.4 (0.8,22.3)		1.6 (0.3,8.4)	
	Divorced	6.5 (0.28,33.4)		2.3 (0.4,12)	
Socioeconomic status	Low	1	0.55	–	
	Moderate	1.1 (0.84,1.44)		–	
	High	0.93 (0.69,1.25)		–	
	Very high	0.93 (0.69,1.24)		–	
Education	Illiterate	1	< 0.01	1	0.39
	Primary school	0.5 (0.4,0.7)		0.7 (0.5,1.02)	
	Secondary school	0.6 (0.4,0.9)		0.8 (0.58,1.2)	
	Diploma and University	0.5 (0.3,0.7)		0.6 (0.45,1.01)	
BMI	Under weight	1	0.6	–	
	Normal	1.36 (0.78,2.37)		–	
	Over weight	1.07 (0.61,1.88)		–	
	Obese	0.93 (0.51,1.7)		–	
Location	Urban	1		1	
	Rural	0.8 (0.6,1.07)	0.19	0.8 (0.6,1.5)	0.1
Chronic kidney disease	No	1		1	
	Yes	3.7 (1.4,9.4)	< 0.01	3.4 (1.3,8.7)	0.02
Hypertension	No	1		1	
	Yes	2.1 (1.7,2.6)	< 0.01	1.6 (1.3–2.1)	< 0.01
Diabetes mellitus	No	1		1	
	Yes	2.8 (2.3,3.5)	< 0.01	2.3 (1.8,2.9)	< 0.01
Fatty liver	No	1		–	
	Yes	0.82 (0.58,1.16)	0.27	–	
Alcohol consumption	No	1		1	
	Yes	1.8 (1.2,2.5)	0.001	1.5 (1.09,2.2)	< 0.01
Cigarette smoking	No	1		1	
	Yes	1.7 (1.3,2.1)	< 0.01	1.2 (0.9,1.6)	0.05
LDL		1.003 (1.002,1.004)	< 0.01	1.003 (1.002,1.003)	< 0.01
TG		1.001 (1.0003,1.002	< 0.01	1.006 (0.99,1.001)	0.4
FBS		1.005 (1.003–1.007)	< 0.01	1.002 (0.99,1.004)	0.08
Hip circumference		0.9 (0.9,0.9)	< 0.01	0.9 (0.9,1.01)	0.1
Waist circumference		1.005 (0.99,1.01)	0.17	–	

^*^P.v for LRT test

All the variables with *p* value less than 0.2 were entered into the multiple Firth Cox regression. Three variables of BMI, socioeconomic status and fatty liver disease were not entered into the multiple model due to having a *p* value higher than 0.2. In addition, waist circumference variable was not included in the multiple model due to its high correlation with hip circumference (*ρ* = 0.8. *p* < 0.0001)

## Discussion

This study estimated the incidence and risk factors of CVDs in adults 40–70 years in Kharameh. In this study, 9442 subjects were followed for 4 years. The density incidence in the present study was estimated to be 6.9 cases per 1000 person-year. In the study of Framingham that conducted by Donald et al., the incidence of CVDs was estimated at 15.7 cases per 1000 person-year [16]. The reason for the difference in incidence density between our study and the study by Donald et al. is probably due to the age difference of the cohort under study; Donald's study included people 50 years and above, and our study was done on 40 to 70-year-old subjects.

In this study, after the age of 60–70 years, chronic kidney disease was identified as the strongest risk factor for CVDs. In another study, in line with the results of the present study, it was stated that kidney dysfunction could double the risk of developing CVDs [17]. Chen et al. also, in their study on people aged 35–65 years who had kidney disease, stated that the risk of developing CVDs in patients with chronic kidney disease was 3.8 times higher than those without it [18]. However, there are several specific factors in chronic kidney patients that may increase the risk of developing CVDs, for example, anemia due to kidney disease, albuminuria, hyperparathyroidism, and oxidative stress [19]. Anemia due to impaired renal function causes left ventricular dysfunction and left ventricular hypertrophy, leading to CVDs and increasing mortality risk fourfold. Other studies have shown that albuminuria plays an important role in the pathogenesis of CVDs and increases the risk of these diseases by 2 to 4 times [19]. Although the rate of CVD is high in those with kidney disease in our study, there were only 31 subjects (0.33% in Table 1), with a total of 4 subjects who eventually developed CVD. However, health policy makers should recognize this to identify and implement appropriate interventions.

Diabetes was also introduced as another risk factor in this study. Dinesh Shah et al. in their cohort study in England on diabetic people over 30 years reported a positive and significant relationship between diabetes and the risk of developing CVDs [20]. Donald and colleagues in the Framingham Cohort Study of people over 50 years of age also identified diabetes as the strongest risk factor for CVDs [16]. In addition, Lee et al. in a cohort study of 2879 men in Singapore reported that the risk of developing CVDs in diabetics was 1.77 times higher than non-diabetics [21]. In Iran, the attributed risk of diabetes for CVDs is reported to be 7.3% [22]. However, many risk factors for diabetes and CVDs are common, such as the role of obesity, age over 45, unhealthy diet, hypertension, stress, and smoking, the role of which cannot be ignored [22]. Furthermore, an unhealthy lifestyle is very common in diabetics, especially the non-elderly and those with academic education [23]. Due to the impact of lifestyle on the incidence of diabetes as a modifiable risk factor for CVDs, health policymakers should develop more up-to-date guidelines to prevent CVDs in individuals with diabetes by modifying their lifestyle [23]. On the other hand, screening for type-2 diabetes is an important strategy to reduce the incidence of CVDs. A study in Denmark showed that screening middle-aged people significantly reduced the risk of all types of CVDs in individuals with diabetes [24].

In the present study, the risk of CVDs in subjects with hypertension was 64% higher than those without it. Donald et al. also in the Framingham Cohort Study of people over 50 years stated that hypertension was significantly associated with the risk of CVDs [16]. A cohort study conducted in Singapore also reported hypertension as the strongest risk factor for CVDs [21]. In another study in Iran, the risk of CVD attributed to hypertension was reported to be 36% [5]. We must remember that hypertension is an important, independent, and modifiable risk factor for CVDs and causes 50% of heart attacks [20]. In Iran, the prevalence of hypertension in individuals aged 40–75 years is estimated at 26.9%. This increase in the prevalence of hypertension is related to changes in individuals' lifestyles, increasing urbanization, and increasing life expectancy [5]. It is, therefore, recommended that the lifestyle should be modified [22].

In this study, the CVDs incidence rate was twice higher in men than in women. In line with the results of our study, another study in south India reported the prevalence of CVDs in men more than women [25]. The prevalence of CVDs in the two sexes is generally different due to several factors, including biological factors and sex hormones, especially estrogens and androgens [26]. The prevalence of CVDs before 50 is higher in men than women and increases in women due to menopause and hormonal changes. In addition, pregnancy-related factors such as diabetes and blood pressure, preeclampsia, and hormonal changes are among the factors that can play a protective role for women [20].

The present study showed that alcohol consumption increased the CVDs incidence rate by 58%. Lee and colleagues in their cohort study in Singapore reported that alcohol played a protective role against CVDs, and stated that the lack of alcohol consumption increased the risk of CVDs 1.8 times [21]. The results of this study were not in the same line with those of the present study. Rehm et al. in their modeling study using WHO data also stated that moderate or low alcohol consumption had no beneficial effect on the risk of CVDs [27]. Another study found that consuming every 30 g of alcohol increased the HDL by 3.66 Mg/dl and Apo Lipoprotein by 8.76 Mg/dl. These factors have a protective role against CVDs [27]. However, we should note that for some reason, including cultural and religious issues in Iran, individuals may not report their alcohol consumption, and there is a probability of underestimation.

In the present study, a significant relationship was seen between the LDL level and risk of developing CVDs. The results of a cohort study in Iran on 8698 people aged 35 to 65 showed that the risk of developing CVDs was 1.54 times higher with increasing levels of LDL [28]. Wilson in a cohort study in Europe and Wallece et al. in their cohort study on 30–74-year-old subjects also reported a direct and significant relationship between LDL levels and the risk of developing CVDs [29, 30]. Two clinical trial studies have shown that the risk of developing CVDs in patients with dyslipidemia is reduced by treating these individuals with a statin drug that reduces dyslipidemia [31, 32].

This study showed a statistically significant relationship between aging and the risk of developing CVDs. In line with the results of our study, Ravi and colleagues stated that the risk of CVD increased with age. They have found that although increasing age has an independent role in the occurrence of CVD, it can be a reflection of the intensity and duration of exposure to other risk factors of CVD [33].

The present study found no statistically significant relationship between BMI and CVDs. However, contrary to the results of our study, overweight and obesity have been suggested as the risk factors for CVDs in many studies [34–37]. On the other hand, in some studies, the obesity paradox is mentioned as an important factor in the relationship between obesity and CVDs [38, 39]. It has also been stated that although obesity increases the risk factors of CVDs and has adverse effects on the structure and function of the heart vessels, obese individuals usually have a better prognosis and less mortality due to CVDs. Lavie and his colleagues have stated several factors for the paradox between obesity and CVDs, such as the presence of protective cytokines in obese individuals, poor response to the renin–angiotensin–aldosterone system, and hypertension in these individuals, which leads to the use of cardiac drugs. In addition, other factors are an increase in body muscle mass and muscle strength, presence of genetic factors, and presence of more metabolic reserves [38].

This study is conducted as the continuation of the cross-sectional study by Baaradeh and his colleagues which investigated the prevalence and risk factors of CVD using the baseline data of the Kharamah cohort [34]. We must state that cross-sectional studies are unable to correctly estimate the causal relationships due to the lack of time sequence. For this reason, there is a need to conduct this cohort study to carefully examine the risk factors. In addition, for accurate intervention planning, in addition to knowing the prevalence rate, we also need the incidence rate. In the present study and a cross-sectional study by Baradeh et al., CVDs were associated with old age, diabetes and hypertension. This is despite the fact that in our study CVDs were associated with alcohol consumption and male sex, but in Baeradeh’s study this relationship was not seen. In addition, in Baeradeh’s study, CVDs were associated with high TGs and smoking, but it was not observed in our study.

### Strengths and limitations

Compared to many other studies, the cohort design and the coverage of a wide range of risk factors are two important strengths of our study. In addition, our study had a large sample size, which increased its generalizability. However, the main limitation of our study was the average duration of the follow-up period (4 years). In addition, we did not have data on the specific types of cardiovascular diseases for each subject. For this reason, we could not calculate the incidence of these diseases separately.

## Conclusion

The present study showed that aging was a risk factor for developing CVDs. In addition, except for age, other identified risk factors are modifiable, such as diabetes, hypertension, and alcohol consumption; however, a large share of cardiovascular events can be reduced by modifying these factors. Therefore, determining interventional strategies and planning to implement appropriate interventions to control and eliminate the risk factors affecting the incidence of CVDs are essential in preventing the occurrence of CVDs. In addition, early disease detection in individuals with risk factors and their control can reduce the risk of CVDs and their burden on the society and individuals.

## Data Availability

The data sets used and/or analyzed during the current study are available from the corresponding author upon reasonable request.

## Abbreviations

CVDs: Cardiovascular diseases
DALYs: Disability adjusted life years
BMI: Body mass index
FBS: Fasting blood sugar
LDL: Low-density lipoprotein
TG: Triglyceride
HDL: High-density lipoprotein
PCA: Principal component analysis
